# Defibrotide (Defitelio): A New Addition to the Stockpile of Food and Drug Administration-approved Oligonucleotide Drugs

**DOI:** 10.1038/mtna.2016.42

**Published:** 2016-08-16

**Authors:** Cy Stein, Daniela Castanotto, Amrita Krishnan, Liana Nikolaenko

**Affiliations:** 1 Department of Medical Oncology and Experimental Therapeutics, City of Hope, Duarte, California, USA; 2 Department of Molecular and Cellular Biology, City of Hope, Duarte, California, USA; 3 Department of Hematologic Oncology, City of Hope, Duarte, California, USA

On 1 April 2016, the Food and Drug Administration approved Defitelio, known for many years as Defibrotide (DF), for marketing in the United States. The indication is severe hepatic veno-occlusive disease (sVOD) following high-dose chemotherapy and autologous bone marrow transplantation, a toxicity of therapy with a high mortality. Defibrotide is not the kind of oligonucleotide drug beloved by molecular biologists and proponents of personalized medicine. Its very complicated mechanism of action, which is still elusive, is without question nonsequence specific, and almost certainly based on the charge-charge interactions of its constituents with biological macromolecules, which are almost certainly proteins.

VOD of the liver, now more commonly known as sinusoidal obstruction syndrome (SOS), is characterized by damage and occlusion of small hepatic venules.^3,4,5^ The pathophysiology of VOD/SOS is not completely understood, but is related to endothelial cell activation by locally released cytokines in the setting of proinflammatory and prothrombotic states during hematopoietic stem cell transplantation (HSCT). The estimated incidence rate of VOD/SOS in patients undergoing HSCT is approximately 10–15% and occurs within 20–30 days of the transplant. Multiple agents can cause endothelial damage; commonly, damage results from the myeloablative conditioning regimen, including chemotherapy or radiation, prior to HSCT. In addition, nontransplant SOS may be caused by liver-directed therapy for the treatment of metastatic cancer, or use of pyrrolizidine alkaloids and other hepato-toxic agents^1,2,3,4,6^ in experimental animals. Severe VOD/SOS is associated with progressive multi-organ failure and a mortality rate of over 80%.VOD/SOS damages endothelial cells, which round up, detach, and eventually occlude the microvascular lumina.^2,8^ Occlusion of the vessel lumina is eventually followed by hepatic stellate cell activation and by deposition of collagen in the hepatic venules,^9^ followed by perivascular hepatocyte necrosis. Sinusoidal obstruction leads to a reduction in hepatic venous outflow and development of postsinusoidal hypertension and further liver damage.^5,6,7^

DF is a polydisperse mixture of single-stranded (90%) and double-stranded (10%) phosphodiester oligonucleotides (length 9-80mer; average 50mer; average molecular mass 16.5 ± 2.5 kDa).^1,2^ It has been known for decades that phosphodiester oligonucleotides are rapidly degraded in plasma. Therefore, it is possible that the active oligomers in DF are those that are double stranded, by virtue of their ability to form intra-strand stem loop structures, or inter-strand concatamers. These higher order structures could provide some measure of nuclease resistance, stabilizing the individual strands for long enough for them to reach the liver, the target of drug activity.

DF cannot be produced by DNA synthesizers. Rather, it is a natural product obtained through the controlled depolymerization of porcine intestinal mucosal DNA. This means that the concentration of any specific sequence in the DF *gemisch* is probably not much greater than the femtomolar range. For this reason alone, DF cannot act via an antisense-type mechanism. It is also well understood that the individual strands that compose DF cannot be resolved by any known physical separation method, including capillary gel electrophoresis.

DF is the only known successful treatment currently available for VOD/SOS. Richardson and colleagues^10^ evaluated effects of DF at an administered dose of 25 mg/kg/day as a treatment for severe VOD post-HSCT in a phase 3 multi-center clinical trial. The study enrolled 102 patients with severe VOD/SOS and multi-organ failure post-HSCT into the DF group. However, this was not a “classical” phase 3 trial as there was no contemporaneous comparator arm. Rather, patients treated with DF were compared to 32 patients in a case-matched historical-control cohort, culled from over 6,880 cases of VOD. The reason the Food and Drug Administration accepted this unusual basis of comparison is also remarkable: It appears that none of the local principle investigators were willing to trust his or her patients to the standard therapy for sVOD, often low molecular weight heparin, but sometimes N-acetylcysteine or ursodeoxycholic acid, as no one believed they were sufficiently active.

The primary endpoint of the trial was patient survival rate at day +100 post-HSCT, with 38.2% observed in the DF group and 25% in the control group. The secondary endpoint was the complete response rate (*i.e.*, complete resolution of all signs and symptoms attributable to sVOD), with a 25.5% rate observed in the DF-treated cohort and a 12.5% rate in the control group. These results also demonstrated a significant improvement in day +100 survival and in complete response rates in patients treated for severe VOD/SOS with DF. The reported adverse events with the use of DF included hemorrhagic events and hypotension.^5,6,7,10,11^

Over the years, we and others have demonstrated that DNA oligomers can, in at least some ways, mimic several of the features of heparin, because both are polyanions. Most of this work with DNA oligomers has been performed with phosphorothioate (PS) oligomers, though in principle, any protein that binds PS oligomers will also bind phosphodiester oligomers, albeit with lesser affinity. The proteins that bind phosphodiester oligomers are also heparin-binding proteins. These proteins and DF appear to interact predominately through charge-charge interactions: The negative charge on the DNA oligomers binds through ionic interactions with swaths of positive charge on the heparin-binding protein. Collagen I, for example, is a basic protein due to its numerous lysine residues. It also binds DF with relatively high affinity. The presence of the nucleobases in the DF strands is also critical for high-affinity binding: They appear to provide a degree of rigidity to the strands and to limit their extent of rotational freedom.

A study of the interactions of DF with heparin-binding proteins was performed by Benimetskaya *et al*.^2^, who determined the Michaelis-Menton binding constants for each interaction. These included vascular endothelial growth factor (VEGF)165 (*K*_M_ = 34 µmol/l); FGF2 (*K*_M_ = 0.5 µmol/l); PDGF BB (*K*_M_ = 5 µmol/l); and collagen I (*K*_M_ = 0.6 µmol/l), respectively. Given the relatively high affinity of DF for VEGF165, it was predicted that the mechanism of action of DF would be independent of VEGF165. This was confirmed by independent contemporaneous observations.^12^ Other heparin-binding proteins, such as tumor necrosis factor-α and HB-EGF, interacted only very weakly with DF.

FGF2 (formerly known as basic FGF) is an important proangiogenic protein that has long been known to promote microvessel formation.^2,13,14,15^ Angiogenesis induction by FGF2 may be direct or indirect, as addition of this growth factor to endothelial cells^16^ results in expression of VEGF, which is also highly proangiogenic.

Due to the ability of DF to bind FGF2, it was capable of releasing ^125^I-FGF2 (but not ^125^I-VEGF165) from its low-affinity binding sites on extracellular matrix. On the other hand, DF did not release ^125^I-FGF2 from high affinity, low picomolar affinity cell surface receptors. This is significant because older data^17,18,19,20^ has shown that mobilization of FGF2 bound to extracellular matrix can promote endothelial cell proliferation. At the same time, DF does not block the binding of FGF2 to its high-affinity cell surface receptors.

In fact, precisely, the opposite situation pertains. Heparin forms a bridge between FGF2 and its cell surface receptors, increasing receptor-ligand affinity and stabilizing the interaction between them. DF was able to substitute for heparin, as both potentiated the proliferative effects of FGF2 on endothelial cells. This was demonstrated in mouse BAF3 cells that were engineered to express the FGFR1 IIIC receptor, to which FGF2 binds with high affinity.^2,21^ DF approximately quadrupled the proliferation of the BAF-3 cells in the presence of FGF2. DF also protected FGF2 from enzymatic (trypsin and chymotrypsin) digestion and air oxidation, but could not inhibit the activity of matrix metalloproteases.^22^ This may be of considerable importance as hepatic cell necrosis, with subsequent protease release, can occur in sVOD.^2,23,24^ DF could also promote the growth of human vascular endothelial cells (HUVECs) both on plastic and underneath collagen I gels. In 3D-collagen I gels, DF stimulated both the proliferation and a dramatic increase (six- to sevenfold) in the tubular morphogenesis of human microvascular endothelial cells-1 (HMEC cells).

However, as stated above, the mechanism of DF is complex, controversial, and not entirely understood. A study by Palomo et al.^25^ investigated the interaction of DF and endothelial cells. The authors showed that the DF uptake in these cells was concentration, time, and temperature dependent. However, these observations could not be extended to other cell types.^25^ Furthermore, the authors showed that the interaction of DF with the cell membrane was sufficient to produce its anti-inflammatory and antioxidant effects, and that its uptake did not require the involvement of adenosine receptors. This contradicts previous observations,^26,27^ underlining the complexity of the mechanism of action of DF.

As mentioned previously, Benimetskaya et al.^2^, demonstrated that DF binds to and protects FGF2, which in turn stimulates endothelial cell mitogenesis. Endothelial tubular morphogenesis was also promoted. Therefore, in the experimental systems employed by these authors, DF seemed to promote angiogenesis.^2^ However, it is also plausible that DF's proangiogenesis activity is at least in part a result of an antagonistic action on the apoptotic pathway. Consistent with this possibility is a study,^28^ that demonstrated the antiapoptotic effects of DF on fludarabine-treated HMECs, and its ability to downregulate the cytotoxic T-lymphocyte response against endothelial cells.^28^ The observation that DF can also display the opposite behavior by demonstrating antiangiogenic potential,^12^ also emphasizes that the action of this drug is probably cell/system and concentration dependent.^25^ Of note, the antiangiogenic activity detected in HUVEC and HMEC cells^12^ seems to develop into proangiogenic (and/or antiapoptotic) activity at an approximately fourfold higher concentration in the identical cell types.^2^

But the mechanism of action of DF is far more complex than noted above, or than that that can be described in the space allowed here. The reader is referred to an excellent review by Ferrero and colleagues,^1^ in which many of the other activities of DF are discussed. In brief, over the years, it has been appreciated that DF is potently antithrombotic^1,2^ and fibrinolytic.^1,29^ DF increases plasma tissue plasminogen activator activity, and decreases the activity of its inhibitor (PAI-1). It can also release tissue-factor pathway inhibitor from endothelial cells,^30^ and inhibit platelet aggregation by increasing the plasma concentration of prostaglandin E2.^31^ All of these effects of DF, and many others described by Pescador et al.,^1^ may be anticoagulating at the site where DF concentrations are highest and where DF is needed most—at the hepatic sinusoidal endothelium.

So is the fundamental mechanism of severe VOD coagulopathy, or is it obstruction by endothelial cells, as suggested by DeLeve *et al*., or is it a combination of both, and much else besides? Regardless, DF is an approved oligonucleotide drug that is well tolerated by patients and it is the best and, thus far the only choice to treat sVOD/SOS.

## References

[bib1] Pescador, R, Capuzzi, L, Mantovani, M, Fulgenzi, A and Ferrero, ME (2013). Defibrotide: properties and clinical use of an old/new drug. Vascul Pharmacol 59: 1–10. 23680861 10.1016/j.vph.2013.05.001

[bib2] Benimetskaya, L, Wu, S, Voskresenskiy, AM, Echart, C, Zhou, JF, Shin, J et al. (2008). Angiogenesis alteration by defibrotide: implications for its mechanism of action in severe hepatic veno-occlusive disease. Blood 112: 4343–4352. 18711003 10.1182/blood-2008-04-149682

[bib3] Valla, DC and Cazals-Hatem, D (2016). Sinusoidal obstruction syndrome. Clin Res Hepatol Gastroenterol (epub ahead of print). 10.1016/j.clinre.2016.01.006 27038846

[bib4] Dalle, JH and Giralt, SA (2016). Hepatic veno-occlusive disease after hematopoietic stem cell transplantation: risk factors and stratification, prophylaxis, and treatment. Biol Blood Marrow Transplant 22: 400–409. 26431626 10.1016/j.bbmt.2015.09.024

[bib5] Fan, CQ and Crawford, JM (2014). Sinusoidal obstruction syndrome (hepatic veno-occlusive disease). J Clin Exp Hepatol 4: 332–346. 25755580 10.1016/j.jceh.2014.10.002 PMC4298625

[bib6] Mohty, M, Malard, F, Abecassis, M, Aerts, E, Alaskar, AS, Aljurf, M et al. (2015). Sinusoidal obstruction syndrome/veno-occlusive disease: current situation and perspectives-a position statement from the European Society for Blood and Marrow Transplantation (EBMT). Bone Marrow Transplant 50: 781–789. 25798682 10.1038/bmt.2015.52 PMC4456788

[bib7] Dignan, FL, Wynn, RF, Hadzic, N, Karani, J, Quaglia, A, Pagliuca, A et al.; Haemato-oncology Task Force of British Committee for Standards in Haematology; British Society for Blood and Marrow Transplantation. (2013). BCSH/BSBMT guideline: diagnosis and management of veno-occlusive disease (sinusoidal obstruction syndrome) following haematopoietic stem cell transplantation. Br J Haematol 163: 444–457. 24102514 10.1111/bjh.12558

[bib8] DeLeve, LD, Ito, Y, Bethea, NW, McCuskey, MK, Wang, X and McCuskey, RS (2003). Embolization by sinusoidal lining cells obstructs the microcirculation in rat sinusoidal obstruction syndrome. Am J Physiol Gastrointest Liver Physiol 284: G1045–G1052. 12584111 10.1152/ajpgi.00526.2002

[bib9] DeLeve, LD, Shulman, HM and McDonald, GB (2002). Toxic injury to hepatic sinusoids: sinusoidal obstruction syndrome (veno-occlusive disease). Semin Liver Dis 22: 27–42. 11928077 10.1055/s-2002-23204

[bib10] Richardson, PG, Riches, ML, Kernan, NA, Brochstein, JA, Mineishi, S, Termuhlen, AM et al. (2016). Phase 3 trial of defibrotide for the treatment of severe veno-occlusive disease and multi-organ failure. Blood 127: 1656–1665. 26825712 10.1182/blood-2015-10-676924 PMC4817309

[bib11] Cheuk, DK, Chiang, AK, Ha, SY and Chan, GC (2015). Interventions for prophylaxis of hepatic veno-occlusive disease in people undergoing haematopoietic stem cell transplantation. Cochrane Database Syst Rev 5: CD009311. 26017019 10.1002/14651858.CD009311.pub2 PMC10891422

[bib12] Koehl, GE, Geissler, EK, Iacobelli, M, Frei, C, Burger, V, Haffner, S et al. (2007). Defibrotide: an endothelium protecting and stabilizing drug, has an anti-angiogenic potential *in vitro* and in vivo. Cancer Biol Ther 6: 686–690. 17495522 10.4161/cbt.6.5.3959

[bib13] Akimoto, T and Hammerman, MR (2003). Fibroblast growth factor 2 promotes microvessel formation from mouse embryonic aorta. Am J Physiol Cell Physiol 284: C371–C377. 12388106 10.1152/ajpcell.00193.2002

[bib14] Bikfalvi, A, Klein, S, Pintucci, G and Rifkin, DB (1997). Biological roles of fibroblast growth factor-2. Endocr Rev 18: 26–45. 9034785 10.1210/edrv.18.1.0292

[bib15] Basilico, C and Moscatelli, D (1992). The FGF family of growth factors and oncogenes. Adv Cancer Res 59: 115–165. 1381547 10.1016/s0065-230x(08)60305-x

[bib16] Seghezzi, G, Patel, S, Ren, CJ, Gualandris, A, Pintucci, G, Robbins, ES et al. (1998). Fibroblast growth factor-2 (FGF-2) induces vascular endothelial growth factor (VEGF) expression in the endothelial cells of forming capillaries: an autocrine mechanism contributing to angiogenesis. J Cell Biol 141: 1659–1673. 9647657 10.1083/jcb.141.7.1659 PMC2132998

[bib17] Jones, LS, Yazzie, B and Middaugh, CR (2004). Polyanions and the proteome. Mol Cell Proteomics 3: 746–769. 15143156 10.1074/mcp.R400008-MCP200

[bib18] Bashkin, P, Doctrow, S, Klagsbrun, M, Svahn, CM, Folkman, J and Vlodavsky, I (1989). Basic fibroblast growth factor binds to subendothelial extracellular matrix and is released by heparitinase and heparin-like molecules. Biochemistry 28: 1737–1743. 2541764 10.1021/bi00430a047

[bib19] Vlodavsky, I, Folkman, J, Sullivan, R, Fridman, R, Ishai-Michaeli, R, Sasse, J et al. (1987). Endothelial cell-derived basic fibroblast growth factor: synthesis and deposition into subendothelial extracellular matrix. Proc Natl Acad Sci USA 84: 2292–2296. 3470794 10.1073/pnas.84.8.2292 PMC304636

[bib20] Baird, A and Ling, N (1987). Fibroblast growth factors are present in the extracellular matrix produced by endothelial cells *in vitro*: implications for a role of heparinase-like enzymes in the neovascular response. Biochem Biophys Res Commun 142: 428–435. 2434094 10.1016/0006-291x(87)90292-0

[bib21] Powers, CJ, McLeskey, SW and Wellstein, A (2000). Fibroblast growth factors, their receptors and signaling. Endocr Relat Cancer 7: 165–197. 11021964 10.1677/erc.0.0070165

[bib22] Lazarus, HM and McCrae, KR (2008). SOS! Defibrotide to the rescue. Blood 112: 3924–3925. 18988876 10.1182/blood-2008-09-177246

[bib23] Kornblum, N, Ayyanar, K, Benimetskaya, L, Richardson, P, Iacobelli, M and Stein, CA (2006). Defibrotide, a polydisperse mixture of single-stranded phosphodiester oligonucleotides with lifesaving activity in severe hepatic veno-occlusive disease: clinical outcomes and potential mechanisms of action. Oligonucleotides 16: 105–114. 16584299 10.1089/oli.2006.16.105

[bib24] Richardson, P and Guinan, E (2001). Hepatic veno-occlusive disease following hematopoietic stem cell transplantation. Acta Haematol 106: 57–68. 11549778 10.1159/000046590

[bib25] Palomo, M, Mir, E, Rovira, M, Escolar, G, Carreras, E and Diaz-Ricart, M (2016). What is going on between defibrotide and endothelial cells? Snapshots reveal the hot spots of their romance. Blood 127: 1719–1727. 26755708 10.1182/blood-2015-10-676114 PMC4817313

[bib26] Bianchi, G, Barone, D, Lanzarotti, E, Tettamanti, R, Porta, R, Moltrasio, D et al. (1993). Defibrotide, a single-stranded polydeoxyribonucleotide acting as an adenosine receptor agonist. Eur J Pharmacol 238: 327–334. 8405101 10.1016/0014-2999(93)90864-e

[bib27] Francischetti, IM, Oliveira, CJ, Ostera, GR, Yager, SB, Debierre-Grockiego, F, Carregaro, V et al. (2012). Defibrotide interferes with several steps of the coagulation-inflammation cycle and exhibits therapeutic potential to treat severe malaria. Arterioscler Thromb Vasc Biol 32: 786–798. 22116094 10.1161/ATVBAHA.111.240291 PMC3288196

[bib28] Eissner, G, Multhoff, G, Gerbitz, A, Kirchner, S, Bauer, S, Haffner, S et al. (2002). Fludarabine induces apoptosis, activation, and allogenicity in human endothelial and epithelial cells: protective effect of defibrotide. Blood 100: 334–340. 12070045 10.1182/blood.v100.1.334

[bib29] Echart, CL, Graziadio, B, Somaini, S, Ferro, LI, Richardson, PG, Fareed, J et al. (2009). The fibrinolytic mechanism of defibrotide: effect of defibrotide on plasmin activity. Blood Coagul Fibrinolysis 20: 627–634. 19809307 10.1097/MBC.0b013e32832da1e3

[bib30] Cella, G, Sbarai, A, Mazzaro, G, Motta, G, Carraro, P, Andreozzi, GM et al. (2001). Tissue factor pathway inhibitor release induced by defibrotide and heparins. Clin Appl Thromb Hemost 7: 225–228. 11441984 10.1177/107602960100700308

[bib31] Coccheri, S, Biagi, G, Legnani, C, Bianchini, B and Grauso, F (1988). Acute effects of defibrotide, an experimental antithrombotic agent, on fibrinolysis and blood prostanoids in man. Eur J Clin Pharmacol 35: 151–156. 3191933 10.1007/BF00609244

